# Sex-Stratified Genetic Analyses Mapping the Influences of Sedentary Behaviors and Physical Activity on Female Reproductive Health

**DOI:** 10.34133/research.1131

**Published:** 2026-02-16

**Authors:** Chongwen Shao, Qian Yang, Lanhui Huang, Haoyu Liu, Qinling Zhu, Xinyi Dong, Hengyu Guan, Xuejiao Bian, Yunfei Huang, Yun Sun

**Affiliations:** ^1^ Department of Reproductive Medicine, Shanghai Key Laboratory for Assisted Reproduction and Reproductive Genetics, Ren Ji Hospital, Shanghai Jiao Tong University School of Medicine, Shanghai, China.; ^2^ MRC Integrative Epidemiology Unit, University of Bristol, Bristol, UK.; ^3^ Department of Geriatric Endocrinology and Metabolism, The First Affiliated Hospital of Guangxi Medical University, Nanning, Guangxi, China.; ^4^ Shandong Provincial Key Laboratory of Animal Cell and Developmental Biology, School of Life Sciences, Advanced Medical Research Institute, Shandong University, Qingdao, Shandong, China.

## Abstract

Female reproductive disorders influence women’s physical and mental well-being, and the sustainability of the family. However, it is unclear whether lifestyle factors, including sedentary behaviors and physical activity, could impact them. Here**,** we performed sex-stratified genome-wide association studies of 5 lifestyle factors in European participants from UK Biobank, including self-reported leisure screen time (LST), sedentary behaviors at work and during commuting, time spent in driving, and daily physical activity. We identified 18 novel sex-stratified variants associated with these exposures and showed that LST was more heritable in women (8.6%) than in men (6.9%). Mendelian randomization (MR) analyses using women-related instruments on 4 female reproductive outcomes demonstrated that per 1-h longer LST a day was robustly associated with elevated risks of menorrhagia (odds ratio [OR] = 1.31, 95% confidence interval [CI] = 1.10 to 1.55), endometriosis (OR = 1.34, 95% CI = 1.11 to 1.60), polycystic ovarian syndrome (OR = 3.05, 95% CI = 1.65 to 5.65), and ectopic pregnancy (OR = 1.35, 95% CI = 1.11 to 1.65), while little evidence was found for sedentary behaviors at work or during commuting. Mediation MR showed that the associations with these outcomes were independent from body mass index, except polycystic ovarian syndrome. Nonlinear MR suggested that LST ≥4 h/day was robustly associated with increased risks of endometriosis and menorrhagia. The findings were independently validated using additional genetic data from the European population and survey data from the National Health and Nutrition Examination Survey. In summary, a simple and actionable lifestyle modification, sitting less, is likely to have independently beneficial influences on multiple reproductive disorders.

## Introduction

Female reproductive health is a fundamental pillar of human well-being and societal sustainability [1]. Menorrhagia represents the underlying reproductive dysfunction and causes disturbances of social life [2], while endometriosis and polycystic ovary syndrome (PCOS) are the most prevalent conditions reducing the possibility of being pregnant [3–6]. Ectopic pregnancy has an incidence of 11/1,000 pregnancies in the UK [7], ends up with a termination of pregnancy, and can be a direct cause of maternal mortality [8]. Prevention of these disorders through modifiable lifestyle risk factors has important clinical implications.

Sedentary behavior and physical activity are 2 main lifestyle factors. Sedentary time was observed to be higher in men than in women [9,10]. Physical activity also shows a consistent sex difference globally, with men being more physically active than women [11]. Measurements of sedentary behaviors usually include leisure screen time (LST), sedentary behavior at work (SDW) and during commuting (SDC), and time spent in driving (TSD). A previous sex-stratified meta-analysis of genome-wide association study (GWAS) of sedentary behaviors and physical activity neither reported the signals they identified by sex nor conducted post-GWAS comparisons between sexes [12]. Thus, it remains unclear whether genetic effects on sedentary behaviors are different between women and men.

Associations of sedentary behaviors and physical activity with female reproductive complications are not equally investigated. A recent systematic review included 4 sedentary-related observational studies (number of participants ranging from 84 to 796), among which unfavorable associations of sedentary behaviors with PCOS were shown in 2 studies, while null associations were found in the other 2 studies [13]. However, these studies are susceptible to residual confounding from factors such as socioeconomic status and other lifestyle variables [14], leading to inconclusive results. Regarding physical activity, randomized controlled trials demonstrated that regular exercise improves menstrual regularity and fertility in women with PCOS [15,16]. However, evidence for other reproductive health outcomes was limited.

Mendelian randomization (MR) provides an approach to assess the causal effects of sedentary behaviors and physical activity on female reproductive disorders, ideally by using female-related genetic variants as instruments. MR is less susceptible to confounding than conventional observational studies, because genetic variants are randomly allocated at meiosis and rarely be influenced by sociodemographic or other lifestyle factors [17]. In a 2-sample MR, the variant–exposure and variant–outcome associations can come from independent studies within the same underlying population [18]. Recent MR studies took advantages of the availability of GWAS data, and found a null association of LST with PCOS but inconsistent associations with endometriosis [19,20]. However, these studies made a strong and potentially implausible assumption that the variant–LST associations were the same in men and women, which is unclear in reality. Therefore, it is still unknown whether the sex-specific variants for LST affect these reproductive disorders.

**Fig. 1.**
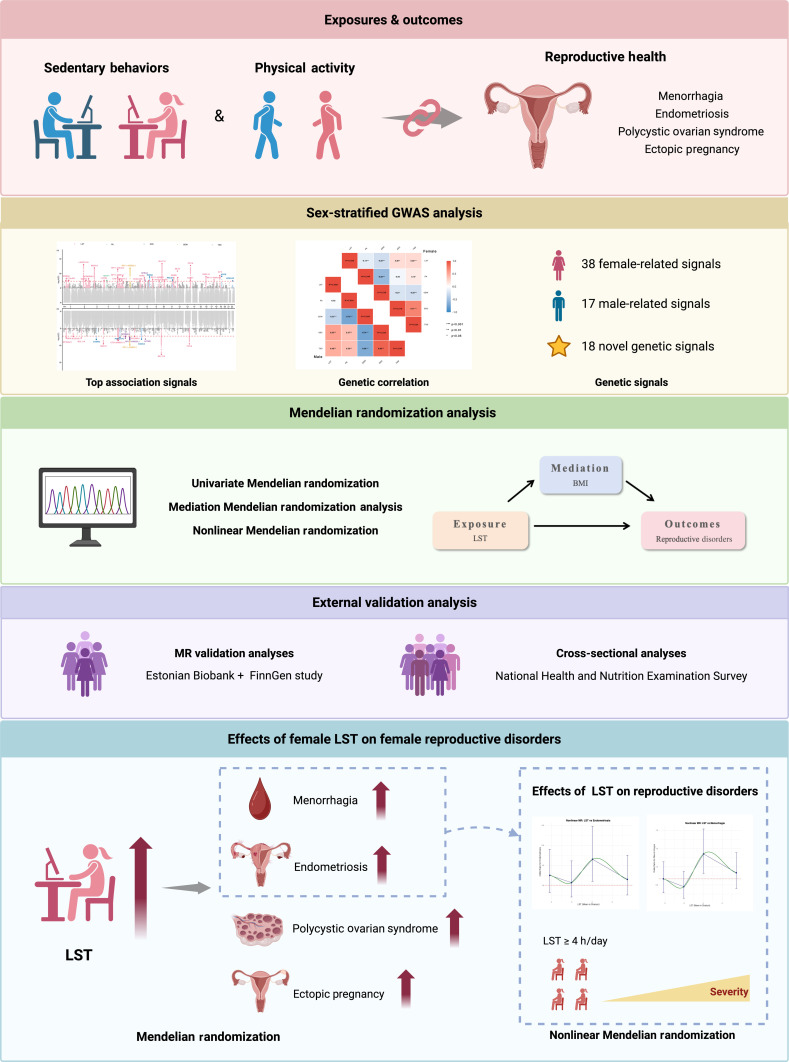
Design of the study. Abbreviations: GWAS, genome-wide association studies; LST, leisure screen time; BMI, body mass index. Created in BioRender.

The aim of our study is to (a) explore the sex difference in genetic effects, heritability, and genetic correlations of 4 sedentary behaviors (LST, SDW, SDC, and TSD) and daily physical activity (DPA); (b) study the associations of these 5 lifestyle factors with 4 reproductive disorders in women, including menorrhagia, endometriosis, PCOS, and ectopic pregnancy; (c) investigate whether these associations were mediated by body mass index (BMI); and (d) inform the identification of LST threshold on improving reproductive health (see Fig. 1). We followed STROBE-MR (Strengthening the Reporting of Observational Studies in Epidemiology Using Mendelian Randomization) guidelines to report this study [21].

## Results

### Characteristics of UK Biobank participants

A total of 502,154 individuals were obtained from the UK Biobank (UKB). Among them, 39,755 were excluded due to non-European descent or failure to pass genetic quality control. A total of 462,399 individuals of European ancestry were included in this study. Among them, men showed a higher average value of LST compared to women (averagely 3.67 h/day for women and 4.10 h/day for men) and a higher average value of DPA compared to women (7.64 h/day for women and 8.98 h/day for men).

### Sex-stratified genetic associations of sedentary behaviors and physical activity

**Fig. 2.**
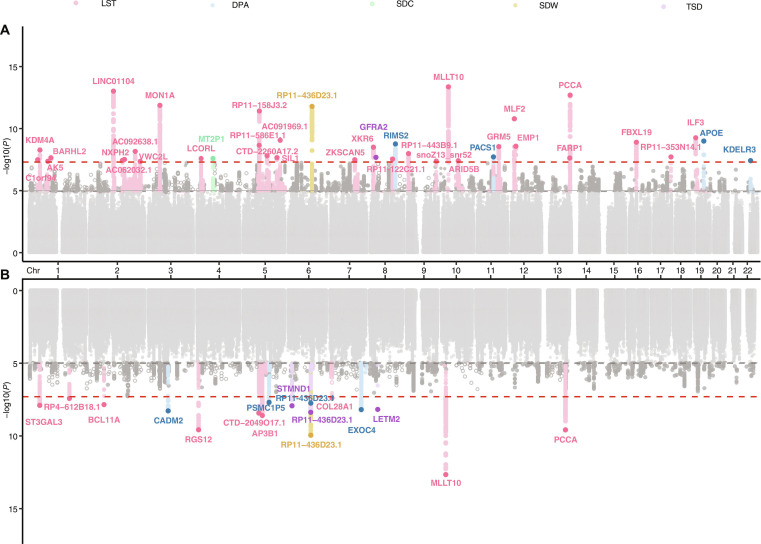
Sex-stratified genome-wide association signals of sedentary behaviors and physical activity. Manhattan plot shows (A) female and (B) male genetic variants independently associated with sedentary behaviors and physical activity at *P* < 5 × 10^−8^. Top genetic variants were mapped to the nearest gene and were highlighted in this plot.

We conducted sex-stratified GWASs of LST, SDW, SDC, TSD, and DPA in UKB participants of European ancestry (*N* = 462,399) (Table S1) by using REGENIE version 4.1 [22]. The linkage disequilibrium score regression (LDSC) [23] intercepts showed almost no evidence to support genomic inflation for these GWASs. For LST, the sex-stratified GWAS identified 1,364 female and 387 male genetic variants reaching genome-wide association signals of 5 × 10^−8^. We identified 31 and 9 independent genetic variants associated with LST in women and men using GCTA-COJO [24], respectively (Table S2). These genetic signals were located in 40 genomic regions. The top hits for both women (rs6482188) and men (rs12251016) were inside the *MLLT10* region (Fig. 2). For the 40 top variants, 34 of them have been reported previously in Europeans. Five female loci (*C1orf94, BARHL2, NXPH2, RP11-586E1.1,* and *ARID5B*) and one male locus (*COL28A1*) were novel association signals (Table S2). Among the top association signals, 8 of them were likely to be functional in an epigenetic regulation point of view (RegulomeDB rank = 1; Table S2).

For the other 4 behavior phenotypes, 15 variants achieved the conventional genome-wide significance threshold (2 for SDW, 1 for SDC, 4 for TSD, and 8 for DPA), 12 of which were novel genetic signals that were not reported previously (Fig. 2 and Table S3). For 15 variants associated with the other 4 behavior phenotypes, one of them was likely to be functional (RegulomeDB rank = 1; Table S3).

We further estimated genome-wide SNP-heritability (*H*^2^) for the 5 exposures using LDSC. LST showed higher heritability in women (*H*^2^ = 8.6%, SE = 0.004) than in men (*H*^2^ = 6.9%, SE = 0.004), while the heritability of the other 4 lifestyle traits was higher in men than in women. The pairwise *Z* score test suggested that the heritability was statistically different between women and men for LST (*Z* = 3.07, *P* value = 0.002), SDW (*Z* = −2.71, *P* value = 0.007), and TSD (*Z* = −3.76, *P* value < 0.001) (Table S4).

**Fig. 3.**
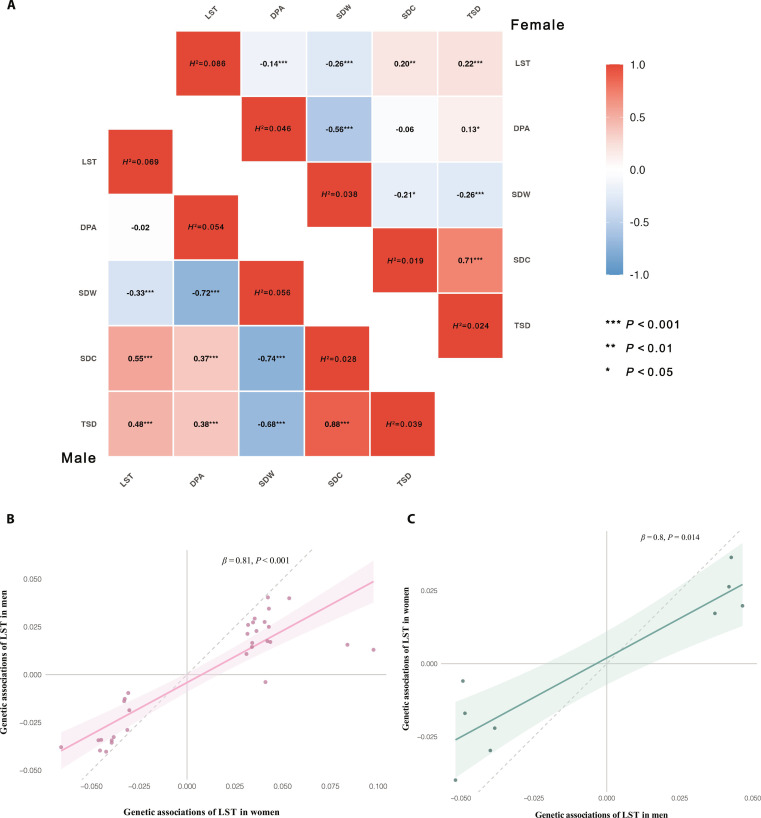
Genetic correlation and sex-specificity correlations of exposures. (A) Genetic correlation of sedentary behaviors and physical activity. (B) Correlations of 31 female-specific genetic signals between female and male LST. (C) Correlations of 9 male-specific genetic signals between female and male LST. The smoothing lines and the shaded regions illustrate the general trend and the 95% confidence intervals (CIs) for the correlation estimates. The Spearman's correlation coefficient (*ρ*) and *P* was employed to quantify the correlation.

Sex-stratified genetic correlations among the 5 traits exhibited moderate to strong correlations (Fig. 3A and Table S5). In general, women tended to have lower genetic correlations among these exposures compared to men. LST showed positive genetic correlations with SDC and TSD, and negative genetic correlations with DPA and SDW, with consistent patterns between sexes. In addition, SDC and TSD showed high genetic correlations between each other, suggesting a shared genetic basis between them.

Given the relatively high heritability of LST and the good number of robust LST-associated variants, we further estimated Spearman’s correlation between female- and male-related genetic signals for LST. The 31 female-specific signals (Spearman's correlation [*ρ*] = 0.81, *P* < 0.001) and the 9 male-specific signals (*ρ* = 0.80, *P* = 0.014) both showed strong correlations between genders (Fig. 3B and C**)**. Estimates for the effect sizes of sex-specific variants were stronger in the corresponding genders, which means it is necessary to use the sex-stratified genetic variants for the following female-specific MR analyses.

### Effects of female-specific exposures on female reproductive disorders

The variant-specific *F*-statistics for female-related LST instruments ranged from 29.96 to 57.00, suggesting sufficient instrument strength (Table S2). Given the genetic similarity between SDC and TSD and the limited number of instruments for TSD, we used SDC to represent both traits. SDW, SDC, and DPA all showed sufficient instrument strength (*F*-statistic >10) after including variants associated with these traits at *P* < 1 × 10^−5^ (Table S6A to C).

**Fig. 4.**
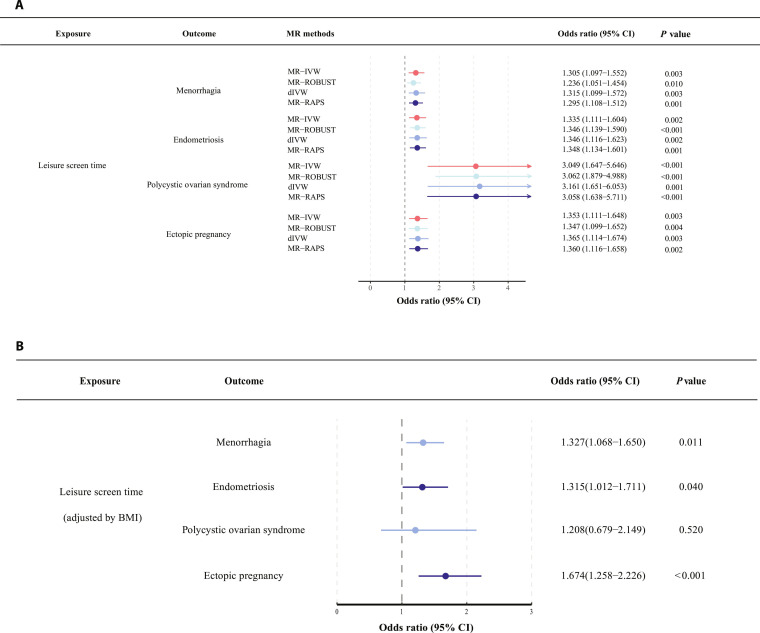
Mendelian randomization estimates of leisure screen time on reproductive health disorders and the effects adjusted for body mass index. (A) Mendelian randomization estimates of leisure screen time on reproductive health disorders. (B) Mediation analysis estimates of leisure screen time adjusted by body mass index on reproductive disorders. Abbreviation: MR-IVW, Mendelian randomization-inverse variance weighted; MR-RAPS, Mendelian randomization-robust adjusted profile score; dIVW, debiased inverse-variance weighted.

After performing MR with 4 reproductive disorders as the primary outcome (Table S7), we observed that per hour longer LST increased risks of menorrhagia, endometriosis, PCOS, and ectopic pregnancy by 31%, 34%, 205%, and 35%, respectively (odds ratio [OR] of menorrhagia per hour increase of LST = 1.305, 95% confidence interval [CI] = 1.097 to 1.552; OR of endometriosis = 1.335, 95% CI = 1.111 to 1.604; OR of PCOS = 3.049, 95% CI = 1.647 to 5.646; OR of ectopic pregnancy = 1.353, 95% CI = 1.111 to 1.648) using MR-inverse variance weighted (IVW) method. These results passed the stringent Bonferroni correction. Other MR sensitivity methods, including MR-robust adjusted profile score (RAPS) [25], MR-ROBUST [26], and debiased IVW [27] showed consistent effect estimates with the MR-IVW method (Fig. 4A and Table S8), which enhanced the reliability of our findings.

Using weak instruments, SDW was marginally associated with a lower risk of ectopic pregnancy and a higher risk of endometriosis, but no evidence was found for other outcomes (Table S9). These observed associations, however, were not robust to the adjustment for multiple comparisons. We observed no evidence for the associations of SDC or DPA with all 4 reproductive outcomes (Table S9). Sensitivity analyses using other MR methods showed largely consistent results.

### Mediation effects of LST on female reproductive outcomes through BMI

To further explore whether BMI acts as a mediator in the MR effects of LST on the female reproductive outcomes, we performed a 2-step mediation MR analysis. In the first step, longer LST increased BMI levels (Table S10). In the second step, increasing BMI reduced endometriosis risk and increased PCOS risk. No effect was observed for BMI against either menorrhagia or ectopic pregnancy. Multivariable Mendelian randomization (MVMR) analysis suggested an independent effect of LST on increased risk of menorrhagia, endometriosis, and ectopic pregnancy after adjusting for the BMI effect. The positive effect of LST on PCOS in the univariable MR was attenuated to the null after adjusting for BMI (Fig. 4B and Table S11). Sensitivity analyses using MVMR-Egger, Robust MV-IVW, and MV-weighted median [28–30] showed largely consistent results.

### Nonlinear associations between LST and female reproductive outcomes in UKB

**Fig. 5.**
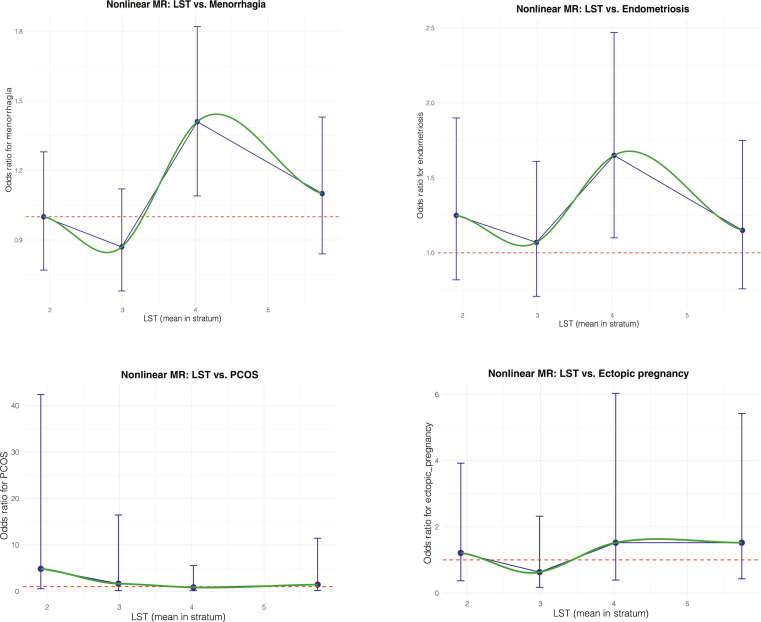
Nonlinear Mendelian randomization of leisure screen time and reproductive disorders. Abbreviations: LST, leisure screen time; PCOS, polycystic ovarian syndrome.

Given the physical and psychological impacts of the 4 reproductive disorders, we aimed to identify a sedentary time threshold, below which the likelihood of developing these reproductive disorders was low. We employed a doubly ranked model for the nonlinear MR (NLMR) approach [31,32] and found nonlinear effects of LST on both menorrhagia and endometriosis, particularly at 4 h/day, with some overlaps of 95% CIs with other time thresholds (Fig. 5). In terms of PCOS and ectopic pregnancy, our NLMR results had very wide 95% CIs in each stratum (Fig. 5), suggesting limited numbers of cases and thus insufficient statistical power for these 2 outcomes.

We used age and hair color as negative control outcomes for our NLMR analyses. These variables were chosen because they are unlikely to be causally related to LST. Our results showed no evidence to support the effect of LST on age or hair color in any stratum (Table S12), enhancing the credibility of our NLMR results for reproductive outcomes.

### Falsify Mendelian randomization assumptions

We report findings according to the STROBE-MR guidelines (Table S13). To ensure the validity of our MR findings between female LST and the tested reproductive health disorders, we tested the following core MR assumptions:

For relevance assumption, we evaluated the strength of the instruments by checking the *F*-statistics. The *F*-statistics for all exposures were greater than 19.49 (Table S2 and S6A to C), indicating that the instruments are strongly associated with the exposure. MR-ROBUST, MR-RAPS, and dIVW methods were employed in addition to MR-IVW, which were designed to deal with weak instrument bias. We observed similar MR estimates among these methods, which again suggested that weak instrument bias is unlikely to be a key issue for our study.

For exchangeability assumption, the exposure and outcome data were both from European ancestry, although some outcome data were from Finnish ancestry, which have minor departure of population structure compared to the rest of the European population. By checking the allele frequency differences between our exposure and outcome data, we observed no major differences (absolute difference of effect allele frequency between exposure and outcome data <0.1). This implies that population stratification is less likely to be an issue for our findings.

For exclusion restriction, we assessed the potential horizontal pleiotropy using MR-Egger regression. The intercept term from MR-Egger regression was not statistically different from zero (*P* ≥ 0.05). We employed the MR-PRESSO method to identify outlying genetic instruments and recalculated the causal effect estimates after excluding these outliers. Using Cochran’s *Q*, we found the existence of heterogeneous effects of LST on menorrhagia and endometriosis and across instruments. In addition, it is well-known that sex hormones influence many female reproductive health outcomes, which may create potential pleiotropic pathways linking LST with reproductive outcomes. To minimize such possibility of pleiotropy, we included both LST and 5 sex hormones as exposures in a set of multivariable MR models. This analysis showed that (a) LST was not associated with any of these sex hormones (Table S10), and (b) LST showed independent effects on the reproductive outcomes, after adjusting for the effect of the sex hormones (Table S14A to E). Therefore, sex hormones are very unlikely to be pleiotropic factors in this study. To evaluate whether individual variant disproportionately influenced the results, we conducted a leave-one-out sensitivity analysis. We found a stable effect across iterations, indicating that no single SNP was driving the association (Fig. S1).

To verify the directionality of the causal effects, we conducted the Steiger filtering test for instruments [33]. All of them passed this test, as no discrepancies were observed between the exposure and outcome associations (Steiger filtering = False, *P* > 0.05). This implies that the direction of effect is likely to influence the exposure first, and then influence the outcome as a causal consequence.

### External validation

We have conducted 2 sets of validation analyses using independent datasets from multiple resources.

**Fig. 6.**
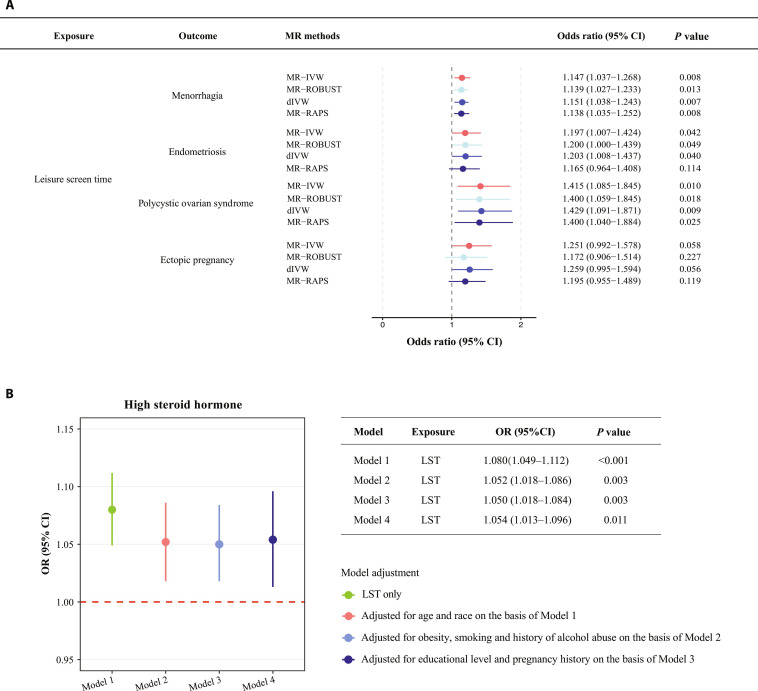
External validation of leisure screen time on reproductive health disorders. (A) Mendelian randomization estimates of leisure screen time on external datasets of reproductive health disorders. (B) Association between LST and high steroid hormone level of NHANES data. Abbreviation: MR-IVW, Mendelian randomization-inverse variance weighted; MR-RAPS, Mendelian randomization-robust adjusted profile score; dIVW, debiased inverse-variance weighted.

First, we conducted an independent validation using a recent GWAS meta-analysis study of reproductive health disorders as outcomes. This study combined 2 cohorts—the Estonian Biobank and the FinnGen study, with up to 293,618 women participants involved [34]. Using the external datasets, we observed consistent associations between LST and female reproductive disorders, including menorrhagia (OR = 1.147, 95% CI: 1.037 to 1.268), endometriosis (OR = 1.197, 95% CI: 1.007 to 1.424), and PCOS (OR = 1.415, 95% CI: 1.085 to 1.845), while ectopic pregnancy showed a trend of elevated risk with 95% CI overlapping with the null (OR = 1.251, 95% CI: 0.992 to 1.578, *P* = 0.058; see Table S15 and Fig. 6A).

Second, we conducted an observational association analysis using data from the United States National Health and Nutrition Examination Survey (NHANES) (Table S16). In the fully adjusted model, per 1-h longer sedentary time was associated with a 5.4% increased risk of high steroid hormone (OR = 1.054, 95% CI = 1.013 to 1.096; Table S17 and Fig. 6B), in which high steroid hormone is a marker for the clinical diagnosis of PCOS [35]. In the other 3 sequential models, we observed the consistent finding that longer sedentary time was also associated with a higher risk of high steroid hormone (Table S17 and Fig. 6B).

The consistency of the MR validation analyses and cross-sectional analyses in the general population confirms the reliability of the core findings derived from our main analysis.

### Investigation of potential mechanisms

Using MELODI-presto [36], we found obesity, gestational diabetes, hypertensive disease, vitamin D deficiency, and inflammasomes as potential intermediate traits linking LST to PCOS, as well as hypertensive disease as a potential intermediate trait linking LST to ectopic pregnancy (Table S18).

## Discussion

Our study estimated the sex-stratified genetic effects of sedentary behaviors and physical activity, and identified 18 novel variants associated with these behaviors. We found that LST is more heritable in women than in men, and per 1-h longer LST in women increased their risks of menorrhagia, endometriosis, PCOS, and ectopic pregnancy by 31%, 34%, 205%, and 35%, respectively. All associations were independent from BMI, emphasizing the key value of intervention on LST to prevent these outcomes, except for PCOS that was mainly mediated through BMI. Collectively, we underlined the importance of considering sedentary behaviors to prevent reproductive disorders and proposed LST <4 h/day as a potential intervention target for reproductive health.

Some of our results were generally consistent with previous evidence. Two previous GWASs have identified a set of genetic signals associated with sedentary behaviors and physical activity [12,37]. One large-scale GWAS meta-analysis of LST has identified 91 genetic signals [12], among which 34 signals were overlapped with the sex-stratified genetic signals found in the current study. Our study reported 38 female-related variants of sedentary behaviors and physical activity in comparison with only 17 male-related variants.

Our unfavorable association of long LST with PCOS was partly consistent with previous evidence. A cross-sectional study of women aged 22 to 27 years (796 cases and 7,051 controls) found that ≥8 h/day sitting time was associated with a higher risk of PCOS, particularly in overweight and obese women [38]. Previous MR studies showed a null association of LST with PCOS [19,20], which is inconsistent with our findings. However, there are fundamental issues in their MR analyses, which would be expected to bias the estimates. First, their instruments for LST were identified in a sex-combined GWAS, assuming women and men have similar genetic effects. In our study, we found that LST is more heritable in women (8.6%) than in men (6.9%), suggesting that the sex-combined approach could be less convincing. Second, their GWAS of PCOS were obtained from FinnGen R5 (642 cases, 118,228 controls, prevalence = 5.15%) released in 2021, even though new versions with more PCOS cases were available. Their prevalence of PCOS was twice lower than that in the general population (10% to 13%) [39], suggesting a potential nondifferential misclassification that would bias the MR results [40]. Our study overcame these issues by using female-related instruments of LST and recently conducted GWAS meta-analysis of PCOS with a prevalence of 8.90% [41]. In addition, our mediation MR results suggested the role of BMI in the causal pathway from LST to PCOS. This aligns with the evidence that PCOS was massively influenced by body weight [42,43]. Future studies focusing on the effect of LST on the subtypes of PCOS among normal-weight individuals could further inform clinical practice.

Our unfavorable association of long LST with menorrhagia was, to some extent, consistent with a previous questionnaire-based study in South Asia, which reported that sedentary behavior was associated with increased risks of menstrual disorders [44]. Our MR findings were directionally consistent with a previous cohort study of 495 Americans, which found a modestly harmful effect of longer sitting time on endometriosis [45]. Meanwhile, one of the above-mentioned MR studies also suggested a harmful effect of longer LST on this outcome [19]. However, compared with this previous MR study, we provided more precise estimates by using female-related instruments and GWAS of endometriosis with more cases. In addition, our MR estimates for the effects of LST on menorrhagia, endometriosis, and ectopic pregnancy were all independent from BMI, highlighting the clinical value of LST as an intervention target for improvement of reproductive health.

To enhance the robustness of our core findings, we performed external validation using independent genetic data from European populations and a population representative survey of US adults. We replicated the main MR findings that LST showed robust associations with menorrhagia, endometriosis, and PCOS in external datasets [34], with effect sizes matching those observed in the main analysis. In the observational analysis, we also observed a robust association between sedentary time and high steroid hormone, implying a consistent association with PCOS. Ectopic pregnancy showed a trend toward increased risk with longer sedentary time. This cross-cohort consistency minimizes the possibility of our findings being driven by cohort-specific biases.

The text-mining analysis provides important clues for revealing pathways through which sedentary behavior affects PCOS and ectopic pregnancy. The identification of obesity, gestational diabetes, hypertensive disease, vitamin D deficiency, and inflammasomes as potential intermediate traits suggests that these factors may play a crucial role, highlighting the metabolic and immune influences in the prevention and management of reproductive health disorders. Future studies could investigate the specific mechanisms underlying these intermediate traits and assess the efficacy of interventions aiming at reducing sedentary behavior to improve reproductive health outcomes.

Our genetic findings identify key loci associated with sex-specific sedentary behavior, which further influence female reproductive disorders, offering actionable insights for targeted interventions [46]. At the population level, these genetic markers can help prioritize high-risk subgroups for tailored public health initiatives [47]. For example, workplace wellness programs can be designed for women of reproductive age with genetic predispositions to sedentary lifestyles. Such targeted efforts optimize the effectiveness of strategies aimed at promoting female reproductive health.

Our findings have important clinical implications. In common practice, women can be actively encouraged to reduce sedentary behavior by strategies such as using standing desks [48], taking regular standing or walking breaks [49], and using health-monitoring reminders [50] to decrease sedentary time. These daily-life activities may improve women’s reproductive health and may also help alleviate symptoms such as dysmenorrhea. Since LST can be easily modified, a decreased LST could potentially reduce risks of reproductive disorders among tens of thousands of women, considering the prevalence of these disorders. However, we acknowledge that our study is just the very first step toward personalized intervention. Our findings need to be further validated in clinical trials before prioritizing them in clinical practice.

There are several limitations. First, our exposures may suffer from measurement errors. We relied on self-reported questionnaires to measure sedentary behaviors and physical activity. Sedentary-time-related measurements mainly included specific behaviors, e.g., watching television, using a computer/screen, or driving. However, there are many other sedentary behaviors in daily life, e.g., sitting while using a phone, which were not captured by our exposure assessment. This exclusion was primarily driven by the technological limitation of UKB baseline data collection period (2006 to 2010). The number of smartphone users in the UK was very low in the total population at that time, where mobile devices were primarily used for basic communication functions rather than prolonged screen-based activities, and thus contributed minimally to overall sedentary time. Nevertheless, this methodological constraint represents an important limitation in interpreting our findings in the modern context. Second, SDW, SDC, and DPA have a limited number of cases and, therefore, did not have enough power to identify sufficient number of instruments achieving genome-wide significance. Thus, we applied a less stringent threshold of 1 × 10^−5^ to select instruments. We estimated the instrument strength and found that the instruments of these traits were over 10, which implies that our instruments are less likely to suffer from weak instrument bias [18,51]. Third, because ectopic pregnancy could hardly be captured by cohorts of young women, we were unable to find literature that reported the associations between sedentary behaviors and ectopic pregnancy. In addition, we conducted analyses using a whole set of MR sensitivity methods that were designed to be robust against weak instruments [25–27]. These methods provided similar results to MR-IVW, which further validated the reliability of our findings. In addition, we were not able to directly test the relevance of genetic instruments for sedentary behaviors (i.e., MR relevance assumption) in women of reproductive age. However, 2 studies demonstrated that sedentary time is stable during young age [52] and the postmenopausal period [53]. A previous study indicated that women tend to maintain stable patterns of sedentary behavior before or during pregnancy and after childbirth [54] and another population representative survey showed that US adults tend to have similar sedentary time among different age strata from 20 to over 65 years old [55]. These papers further support our assumption that sedentary habits may be stable for women during their reproductive age. The current World Health Organization guidelines recommend the general adult population to limit sedentary time, but with limited evidence to suggest a threshold [56]. A meta-analysis of 62 studies collecting self-reported sitting time data showed that adults worldwide sit 4.7 h/day on average [57]. Our study provides evidence to support the reduction of LST to 4 h/day (less than the current daily average) to prevent menorrhagia and endometriosis. Finally, our study was mainly conducted using data from European ancestry. Therefore, the generalizability across ancestries needs to be tested in future studies.

In conclusion, we provide evidence that women showed higher heritability of LST than men. LST showed effects on menorrhagia, endometriosis and PCOS , and tended to show an effect on ectopic pregnancy. We further prioritized 4 h/day of LST as a threshold to prevent endometriosis and menorrhagia, which may inform development of future clinical guidelines of reproductive health.

## Materials and Methods

### Participants of UKB

Our study used individual participant data from UKB. In total, 503,325 adults (~54% women) were recruited into UKB. The collected data encompassed comprehensive metrics including participants’ health profiles, lifestyle patterns, and standardized biometric measurements. Genotyping, pre-imputation quality control, and imputation procedures were described in detail elsewhere [58] and are briefly summarized here. Blood samples were collected at UKB baseline, and genotyped on 2 arrays: the first ~50,000 samples were genotyped on the UK BiLEVE array and the remaining ~450,000 samples were genotyped on the UKB Axiom array. Genotype data were imputed against 2 references panels: the Haplotype Reference Consortium panel and the UK10K + 1000 Genomes panel. We used the imputed data released by UKB in March 2018 and applied in-house post-imputation quality control.

The initial analytical dataset included 502,154 participants. Individual-level exclusions were applied with (a) a sex discordance between genetically inferred sex (data-field 22001) and self-reported sex (data-field 31); (b) putative sex chromosome aneuploidy (data-field 22019); (c) outlier heterozygosity or missing call rates (data-field 22027); (d) a sample-level genotype missing call rate > 0.02; or (e) withdrawal of consent from the UKB. Subsequently, to define a genetically homogeneous sample, individuals of European ancestry were identified using k-means clustering on the first 4 genetic principal components, resulting in a final analytical sample of 462,399 individuals assigned to the European cluster [59].

Variant-level quality control was then implemented via a 2-stage process. In the first stage, variants were required to have a minor allele frequency (MAF) > 0.001, Hardy–Weinberg equilibrium *P* value > 1 × 10^−6^, and variant call missing rate < 0.02. In the second stage, additional filtering of variants was performed based on imputation quality (INFO score) stratified by MAF: INFO > 0.3 for SNPs with MAF > 0.03; INFO > 0.6 for 0.01 < MAF ≤ 0.03; INFO > 0.8 for 0.005 < MAF ≤ 0.01; and INFO > 0.9 for 0.001 < MAF ≤ 0.005.

### Definition of the sedentary behaviors and physical activity measures

Our traits of sedentary behaviors and physical activity were operationalized based on UKB’s standardized assessment protocols, combining objective accelerometer measurements and validated self-reported questionnaires [60]. We defined LST using self-reported information at recruitment via 2 questions: (a) “In a typical DAY, how many hours do you spend watching TV” (data-field 1070) and (b) “In a typical DAY, how many hours do you spend using the computer” (data-field 1080). We derived the LST value by adding the responses to the 2 questions together. We defined SDW using self-reported information at recruitment via the question: “Does your work involve walking or standing for most of the time?” (data-field 806). We defined SDC using self-reported information at recruitment via the question: “What types of transport do you use to get to and from work? (You can select more than one answer)” (data-field 6143). We defined TSD using self-reported information at recruitment via the question: “In a typical DAY, how many hours do you spend driving?” (data-field 1090).

We defined DPA using Total Metabolic Equivalent Task Scores: minutes per week for all activities including walking and moderate and vigorous activities (data-field 22040). Given that the distribution of DPA did not follow a normal distribution, we took the natural logarithm of DPA values for subsequent analyses.

All responses of “do not know” or “prefer not to answer” in the previous questions were coded as missing and thus systematically excluded from the analysis. The detailed information of these phenotypes is listed in Table S1.

### Reproductive health outcomes

To ensure the reliability of our findings, we obtained genetic associations with menorrhagia and PCOS from large-scale GWAS meta-analyses of European ancestry and those with endometriosis and ectopic pregnancy from FinnGen (the latest version R12). Large GWAS meta-analyses of menorrhagia [61] (31,309 cases and 318,510 controls) and PCOS [41] (10,074 cases and 103,164 controls) defined these outcomes by harmonizing self-report data and electronic health records from multiple individual GWAS (Table S7). In FinnGen [62], endometriosis was defined based on ICD-10 codes N80.8 and includes 20,190 cases and 130,160 controls (finngen_R12_N14_ENDOMETRIOSIS). Ectopic pregnancy was defined based on ICD-10 codes O00.1 and includes 7,586 cases and 199,279 controls (finngen_R12_O15_PREG_ECTOP).

As shown in Table S7, we defined menorrhagia, endometriosis, and ectopic pregnancy using the derived variables by UKB for NLMR analysis. These variables integrated information from primary care, hospital inpatient data, the death registry, and participant self-report, to maximize the number of cases. PCOS was defined using ICD-10 code recorded in the hospital inpatient data (Table S7).

### Statistical analyses

#### Sex-stratified GWAS analyses

Sex-stratified GWASs of sedentary behaviors and physical activity were conducted using individual-level data from UKB. The GWAS analysis was conducted using REGENIE version 4.1 [22], to account for population stratification and relatedness. We adjusted for age, biological sex, genotyping array, and the first 10 principal components. These adjustments ensured that the results are robust, and minimized the impact of confounding factors, allowing for accurate estimation of sex-related genetic associations with each phenotype.

We used the GCTA-COJO method [24] to identify conditional independent variants associated with the lifestyle factors. GCTA-COJO was performed based on all variants, except variants without rs-ID, with genome-wide association signals (*P* ≤ 5.0 × 10^−8^) for LST, and suggestive genome-wide association signals (*P* ≤ 1 × 10^−5^) for the other 4 exposures since they did not have enough genome-wide association signals. We selected 10 Mb linkage disequilibrium (LD) distance, and the independent signals were selected through a stepwise selection procedure (i.e., -cojo-slct).

The LDSC method [23] was used to calculate the SNP-heritability using the sex-specific GWAS summary statistics of each trait. The LD estimates from European ancestry samples in the 1000 Genomes projects were used as a reference. The genetic correlation with sedentary behaviors and physical activity were further estimated using bivariate LDSC [23]. The heritability and genetic correlation analysis were conducted using R package ldscr 0.1.0. To estimate the correlations between female- and male-related genetic signals for LST, we conducted Spearman’s rank correlation analysis, a nonparametric statistical method using R package stats version 4.4.1.

GWAS predominantly identifies genetic signals within noncoding genomic regions, which collectively account for over 80% significant associations [63]. To understand the regulatory properties of the independent genetic signals, we used RegulomeDB database v2 to annotate the LST genetic signals. For each locus, the top hit and its LD proxies (with LD *r*^2^ > 0.8 in 1000 Genomes Project Europeans) were included in the annotation.

#### Mendelian randomization analyses

##### Instrument selection

We used a 3-step procedure to select instruments for LST: (a) we removed ambiguous and palindromic variants using the pre-defined approach included in the harmonization step of the TwoSampleMR R package [64] and MendelianRandomization R package [28]; (b) in order to assess the directionality of the causal effect between female LST and reproductive outcomes, we performed the Steiger filtering test [33]. This test evaluates whether the genetic instruments used for the exposure are more strongly associated with the exposure than with the outcome, ensuring that the causal direction is consistent with the assumed model; (c) *F*-statistics were calculated to assess the strength of instruments based on the association between the SNP and the exposure. Higher *F*-statistic values indicate stronger instruments, and an *F*-statistic greater than 10 indicates that the instruments are sufficiently strong and less likely to suffer from weak instrument bias. For LST, we used a *P* value cutoff of 5 × 10^−8^ to select instruments. For SDC, SDW, and DPA, due to relatively low statistical power, we applied a slightly relaxed *P* value cutoff of 1 × 10^−5^ to select instruments. TSD showed insufficient instruments at 1 × 10^−5^. Given its genetic similarity to SDW, we used SDW as a proxy for TSD.

##### Estimation of effects of 4 exposures on reproductive disorders

We conducted 2-sample MR analyses of 4 female-related exposures (instruments identified by the sex-stratified GWAS of LST, SDW, SDC, and DPA) on the 4 reproductive outcomes (menorrhagia, endometriosis, PCOS, and ectopic pregnancy) to detect the possible causal relationship. We harmonized GWAS summary data of each exposure and outcome through allele frequency alignment and strand orientation standardization. The MR-IVW method was used as the discovery approach. A set of sensitivity analyses methods, including MR-RAPS [25], MR-ROBUST [26], and dIVW [27], was applied. We used Bonferroni correction to adjust for multiple comparisons, and the *P* value threshold was 0.0031 (i.e., 0.05/4 exposures/4 outcomes).

As an external validation, we conducted an MR analysis of LST on the 4 reproductive health disorders. The outcome data were derived from a recent GWAS meta-analysis study of up to 293,618 women participants [34].

##### Estimation of the mediation effect through BMI

To further explore whether BMI acts as a mediator in the univariable MR associations, we performed a 2-step mediation MR analysis. In the first step, we explored the association of LST with BMI, and the female-specific GWAS of BMI (*N* = 37) was obtained from the GIANT consortium [65]. In the second step, we applied MVMR [66] to estimate both the direct effect of LST on female reproductive outcomes and its indirect effect through BMI. The MVMR-IVW method was used as the discovery approach. A set of sensitivity analysis methods, including MVMR-Egger, Robust MV-IVW (using random-effects model and robust regression), and MV-Weighted Median, was applied [28–30].

##### Estimation of nonlinear effects of LST on reproductive disorders

We applied a doubly ranked model for the NLMR analysis [31,32] to investigate the causal relationship between LST and 4 reproductive outcomes with univariate MR evidence. First, genetic risk score was calculated for each individual based on genetic variants associated with LST. The derived genetic risk score was combined with phenotype data (including LST and the associated reproductive outcomes) and incorporated into the analysis model. In all analyses, we controlled for potential confounders, including age, genotyping array, and the first 10 principal components. To ensure the robustness and reliability of the results, we also used participants’ age and natural hair color (data-field 1747) as negative control outcomes to detect selection bias [67], when estimating the nonlinear causal relationship between LST and reproductive outcomes. Briefly, we categorized natural hair color into 4 subgroups, i.e., black, dark brown, light brown/red, and blonde.

##### Sensitivity analyses to falsify MR assumptions

MR analysis relies on 3 key assumptions: relevance, exchangeability, and exclusion restriction [68]. To ensure that the genetic instruments fit the relevance assumption, we used the *F*-statistic to evaluate the validity of the selected instruments, and an *F*-statistic greater than 10 suggested sufficient strength (details in the “Instrument selection” section). For exchangeability assumption, the exposure and outcome data were both from European ancestry. For outcome data from FinnGen [62], we checked the allele frequency differences between the exposure and outcome data. The absolute difference of effect allele frequency between exposure and outcome data >0.1 was defined as the existence of minor departure of the population structure.

For exclusion restriction, we assessed horizontal pleiotropy using the MR-Egger regression, which applies weighted linear regression with an unconstrained intercept [69]. The intercept signifies the mean pleiotropic effect and illustrates the average direct impact of a variant on the outcome. To detect potential horizontal pleiotropy, we employed the MR-PRESSO method to identify outlying genetic instruments that may violate the assumption of heterogeneity. Subsequently, causal effect estimates were recalculated after excluding these outliers. We also evaluated heterogeneity using Cochran’s *Q* test [70] to identify outlier variants. To further evaluate whether individual SNPs disproportionately influenced the meaningful univariable MR results, we conducted a leave-one-out sensitivity analysis [64] by systematically excluding each SNP from MR-IVW and recalculating the causal estimates. A stable effect across iterations indicated that no single SNP was driving the association. Given that sex hormones (i.e., luteinizing hormone, progesterone, estradiol, follicle-stimulating hormone, and testosterone) are known to be causally associated with our reproductive outcomes, we applied MVMR to investigate whether those sex hormones were in the potential horizontal pleiotropy pathway from LST to the outcomes [66]. We obtained GWAS of the sex hormones from the GWAS Catalog [61]. The MVMR-IVW method was used as a discovery approach, and a set of sensitivity analyses, including MVMR-Egger, Robust MV-IVW, and MV-Weighted Median, was applied [28–30].

MR analyses were conducted using R packages TwoSampleMR [64] (version 0.6.6), MendelianRandomization [28] (version 0.10.0), MR-PRESSO (version 1.0), and mr.raps (version 0.2) in R (version 4.4.1).

##### Observational analysis using NHANES

NHANES is a nationally representative cross-sectional survey conducted in the United States, encompassing data on demographics, laboratory tests, questionnaires, etc. We investigated the associations between sedentary behavior and high steroid hormone, which is one of the commonly used diagnostic criteria for PCOS [35]. This analysis included 54,716 female participants aged from 18 to 50, with data derived from NHANES 2011 to 2020 cycles.

Sedentary activity was assessed using the variable PAD680 (minutes of sedentary activity) in NHANES, which captures total daily sitting time (excluding sleep) across contexts including school, home, transportation, and leisure. High steroid hormone level was defined as with any 1 of the 3 following measurements that exceeded the women-specific thresholds: (a) total testosterone >47.94 ng/dl; (b) androstenedione >2.25 ng/ml for participants <40 years or >1.9 ng/ml for participants ≥40 years; or (c) free androgen index >7.64% [71].

Covariates incorporated in our analysis comprised age, race, obesity status, history of alcohol abuse, smoking status, educational level, and ever being pregnant. Complete information on covariate definitions and assessment processes is available in Table S16. The k-nearest neighbors algorithm was employed for the imputation of covariates using the R package VIM 6.2.6 [72]. To examine the association between sedentary time and the reproductive outcome, a series of weighted logistic regression models were constructed using the svyglm function in the R package survey to account for the complex sampling design of NHANES. The models were built in a stepwise adjustment manner: Model 1 was the unadjusted model; Model 2 was adjusted for age and race; Model 3 further adjusted for obesity status, smoking status, and history of alcohol abuse on the basis of Model 2; and Model 4, the fully adjusted model, additionally incorporated educational level and ever being pregnant into Model 3.

##### Identification of intermediate traits through MELODI-presto

To explore potential intermediate trait overlap between sedentary behavior and female reproductive health outcomes, we utilized the MELODI-presto tool [36] (https://melodi-presto.mrcieu.ac.uk/). It enables the identification of intermediate traits through literature mining. We constructed literature-based semantic triples for sedentary behavior and 4 female reproductive outcomes, and assigned frequency scores to each triple to identify traits with potential mediating effects.

## Ethical Approval

Ethical approval for UKB was obtained from the North West Multi-centre Research Ethics Committee (ref 11/NW/0382). All individuals gave informed consent at enrollment.

## Data Availability

We used both individual participant data from UK Biobank and publicly available summary statistics. Full summary statistics for our sex-stratified genome-wide association study of LST in UK Biobank will be publicly available via the Omics Harbour platform once the manuscript is accepted for publication. The dataset supporting the individual-level data of this article is available in the UK Biobank repository (http://www.ukbiobank.ac.uk/). GWAS summary statistics of female reproductive health disorders can be accessed from FinnGen via its website (https://www.finngen.fi/en/access_results) or the corresponding GWAS meta-analyses presented in our manuscript. The scripts used to conduct the statistical analyses for this study were shared via the GitHub repository (https://github.com/geneinmylife/LST-PREG-project).

## References

[1] American College of Obstetricians and Gynecologists’ Committee on Practice Bulletins—Gynecology. ACOG practice bulletin no. 200: Early pregnancy loss. Obstet Gynecol. 2018;132(5):e197–e207. 30157093 10.1097/AOG.0000000000002899

[2] Oehler MK, Rees MCP. Menorrhagia: An update. Acta Obstet Gynecol Scand. 2003;82(5):405–422. 12752071 10.1034/j.1600-0412.2003.00097.x

[3] De Ziegler D, Borghese B, Chapron C. Endometriosis and infertility: Pathophysiology and management. Lancet. 2010;376(6742):730–738. 20801404 10.1016/S0140-6736(10)60490-4

[4] Bonavina G, Taylor HS. Endometriosis-associated infertility: From pathophysiology to tailored treatment. Front Endocrinol. 2022;13:1020827. 10.3389/fendo.2022.1020827 PMC9643365 36387918

[5] Palomba S, Piltonen TT, Giudice LC. Endometrial function in women with polycystic ovary syndrome: A comprehensive review. Hum Reprod Update. 2021;27(3):584–618. 33302299 10.1093/humupd/dmaa051

[6] Cooney LG, Dokras A. Beyond fertility: Polycystic ovary syndrome and long-term health. Fertil Steril. 2018;110(5):794–809. 30316414 10.1016/j.fertnstert.2018.08.021

[7] Diagnosis and management of ectopic pregnancy: Green-top guideline no. 21. BJOG. 2016;123(13):e15–e55. 27813249 10.1111/1471-0528.14189

[8] Chong KY, de Waard L, Oza M, van Wely M, Jurkovic D, Memtsa M, Woolner A, Mol BW. Ectopic pregnancy. Nat Rev Dis Primers. 2024;10(1):94. 39668167 10.1038/s41572-024-00579-x

[9] López-Valenciano A, Mayo X, Liguori G, Copeland RJ, Lamb M, Jimenez A. Changes in sedentary behaviour in European Union adults between 2002 and 2017. BMC Public Health. 2020;20(1):1206. 32843022 10.1186/s12889-020-09293-1 PMC7448983

[10] Loyen A, Clarke-Cornwell AM, Anderssen SA, Hagströmer M, Sardinha LB, Sundquist K, Ekelund U, Steene-Johannessen J, Baptista F, Hansen BH, et al. Sedentary time and physical activity surveillance through accelerometer pooling in four European countries. Sports Med. 2017;47(7):1421–1435. 27943147 10.1007/s40279-016-0658-y PMC5488150

[11] Johnson LR. Physical activity differs with sex and age. BMJ. 2019;366: Article l5694. 10.1136/bmj.l5694 31570410

[12] Wang Z, Emmerich A, Pillon NJ, Moore T, Hemerich D, Cornelis MC, Mazzaferro E, Broos S, Ahluwalia TS, Bartz TM, et al. Genome-wide association analyses of physical activity and sedentary behavior provide insights into underlying mechanisms and roles in disease prevention. Nat Genet. 2022;54:1332–1344. 36071172 10.1038/s41588-022-01165-1 PMC9470530

[13] Kazemi M, Kim JY, Wan C, Xiong JD, Michalak J, Xavier IB, Ganga K, Tay CT, Grieger JA, Parry SA, et al. Comparison of dietary and physical activity behaviors in women with and without polycystic ovary syndrome: A systematic review and meta-analysis of 39 471 women. Hum Reprod Update. 2022;28(6):910–955. 35639552 10.1093/humupd/dmac023 PMC9629501

[14] Lawlor DA, Tilling K, Davey G. Triangulation in aetiological epidemiology. Int J Epidemiol. 2016;45(6):1866–1886. 28108528 10.1093/ije/dyw314 PMC5841843

[15] Cowan S, Lim S, Alycia C, Pirotta S, Thomson R, Gibson-Helm M, Blackmore R, Naderpoor N, Bennett C, Ee C, et al. Lifestyle management in polycystic ovary syndrome—Beyond diet and physical activity. BMC Endocr Disord. 2023;23(1):14. 36647089 10.1186/s12902-022-01208-y PMC9841505

[16] Stańczak NA, Grywalska E, Dudzińska E. The latest reports and treatment methods on polycystic ovary syndrome. Ann Med. 2024;56(1):2357737. 38965663 10.1080/07853890.2024.2357737 PMC11229724

[17] Davey Smith G, Hemani G. Mendelian randomization: Genetic anchors for causal inference in epidemiological studies. Hum Mol Genet. 2014;23(R1):R89–R98. 25064373 10.1093/hmg/ddu328 PMC4170722

[18] Zheng J, Baird D, Borges M-C, Bowden J, Hemani G, Haycock P, Evans DM, Davey Smith G. Recent developments in Mendelian randomization studies. Curr Epidemiol Rep. 2017;4(4):330–345. 29226067 10.1007/s40471-017-0128-6 PMC5711966

[19] Fu M. Causal relationship between physical activity and common gynecologic conditions: A two-sample Mendelian randomization study. Am J Transl Res. 2025;17(5):3753–3765. 40535620 10.62347/PGYY9493 PMC12170402

[20] Zhang J, Wang T, Yang P, Miao Y, Ge B, Sun J. Association of sleep traits, physical activity, and sedentary leisure behavior with female reproductive health: A two-sample Mendelian randomization analysis. Int J Women’s Health. 2025;17:497–506. 39995886 10.2147/IJWH.S492065 PMC11849416

[21] Skrivankova VW, Richmond RC, Woolf BAR, Yarmolinsky J, Davies NM, Swanson SA, VanderWeele T, Higgins JPT, Timpson NJ, Dimou N, et al. Strengthening the reporting of observational studies in epidemiology using Mendelian randomization: The STROBE-MR statement. JAMA. 2021;326(16):1614–1621. 34698778 10.1001/jama.2021.18236

[22] Mbatchou J, Barnard L, Backman J, Marcketta A, Kosmicki JA, Ziyatdinov A, Benner C, O’Dushlaine C, Barber M, Boutkov B, et al. Computationally efficient whole-genome regression for quantitative and binary traits. Nat Genet. 2021;53(7):1097–1103. 34017140 10.1038/s41588-021-00870-7

[23] Bulik-Sullivan BK, Loh P-R, Finucane HK, Ripke S, Yang J, Schizophrenia Working Group of the Psychiatric Genomics Consortium, Patterson N, Daly MJ, Price AL, Neale BM. LD score regression distinguishes confounding from polygenicity in genome-wide association studies. Nat Genet. 2015;47(3):291–295. 25642630 10.1038/ng.3211 PMC4495769

[24] Yang J, Ferreira T, Morris AP, Medland SE, Genetic Investigation of ANthropometric Traits (GIANT) Consortium, DIAbetes Genetics Replication And Meta-analysis (DIAGRAM) Consortium, Madden PAF, Heath AC, Martin NG, Montgomery GW, et al. Conditional and joint multiple-SNP analysis of GWAS summary statistics identifies additional variants influencing complex traits. Nat Genet. 2012;44(4):369–375. 22426310 10.1038/ng.2213 PMC3593158

[25] Zhao Q, Wang J, Hemani G, Bowden J, Small DS. Statistical inference in two-sample summary-data Mendelian randomization using robust adjusted profile score. Ann Stat. 2020;48(3):1742–1769.

[26] Rees JMB, Wood AM, Dudbridge F, Burgess S. Robust methods in Mendelian randomization via penalization of heterogeneous causal estimates. PLoS One. 2019;14(9): Article e0222362. 31545794 10.1371/journal.pone.0222362 PMC6756542

[27] Ye T, Shao J, Kang H. Debiased inverse-variance weighted estimator in two-sample summary-data Mendelian randomization. Ann Stat. 2020;49(4):2079–2100. 10.1002/sim.10245 39453381

[28] Yavorska OO, Burgess S. MendelianRandomization: An R package for performing Mendelian randomization analyses using summarized data. Int J Epidemiol. 2017;46(6):1734–1739. 28398548 10.1093/ije/dyx034 PMC5510723

[29] Burgess S, Thompson SG. Multivariable Mendelian randomization: The use of pleiotropic genetic variants to estimate causal effects. Am J Epidemiol. 2015;181(4):251–260. 25632051 10.1093/aje/kwu283 PMC4325677

[30] Rees JMB, Wood AM, Burgess S. Extending the MR-Egger method for multivariable Mendelian randomization to correct for both measured and unmeasured pleiotropy. Stat Med. 2017;36(29):4705–4718. 28960498 10.1002/sim.7492 PMC5725762

[31] Tian H, Mason AM, Liu C, Burgess S. Relaxing parametric assumptions for non-linear Mendelian randomization using a doubly-ranked stratification method. PLOS Genet. 2023;19(6): Article e1010823. 37390109 10.1371/journal.pgen.1010823 PMC10343089

[32] Staley JR, Burgess S. Semiparametric methods for estimation of a nonlinear exposure-outcome relationship using instrumental variables with application to Mendelian randomization. Genet Epidemiol. 2017;41(4):341–352. 28317167 10.1002/gepi.22041 PMC5400068

[33] Hemani G, Tilling K, Davey Smith G. Orienting the causal relationship between imprecisely measured traits using GWAS summary data. PLOS Genet. 2017;13(11): Article e1007081. 29149188 10.1371/journal.pgen.1007081 PMC5711033

[34] Pujol Gualdo N, Džigurski J, Rukins V, Pajuste F-D, Wolford BN, Võsa M, Golob M, Haug L, Alver M, Läll K, et al. Atlas of genetic and phenotypic associations across 42 female reproductive health diagnoses. Nat Med. 2025;31:1626–1634. 40069456 10.1038/s41591-025-03543-8

[35] The Rotterdam ESHRE/ASRM-sponsored PCOS consensus workshop group. Revised 2003 consensus on diagnostic criteria and long-term health risks related to polycystic ovary syndrome (PCOS). Hum Reprod. 2004;19(1):41–47. 14688154 10.1093/humrep/deh098

[36] Elsworth B, Gaunt TR. MELODI presto: A fast and agile tool to explore semantic triples derived from biomedical literature. Bioinformatics. 2021;37(4):583–585. 32810207 10.1093/bioinformatics/btaa726 PMC8088324

[37] Chen J, Ruan X, Fu T, Lu S, Gill D, He Z, Burgess S, Giovannucci EL, Larsson SC, Deng M, et al. Sedentary lifestyle, physical activity, and gastrointestinal diseases: Evidence from mendelian randomization analysis. EBioMedicine. 2024;103: Article 105110. 38583262 10.1016/j.ebiom.2024.105110 PMC11004085

[38] Tay CT, Moran LJ, Harrison CL, Brown WJ, Joham AE. Physical activity and sedentary behaviour in women with and without polycystic ovary syndrome: An Australian population-based cross-sectional study. Clin Endocrinol. 2020;93(2):154–162. 10.1111/cen.14205 32324293

[39] Teede HJ, Tay CT, Laven J, Dokras A, Moran LJ, Piltonen TT, Costello MF, Boivin J, M Redman L, A Boyle J, et al. Recommendations from the 2023 international evidence-based guideline for the assessment and management of polycystic ovary syndrome. Fertil Steril. 2023;120(4):767–793. 37589624 10.1016/j.fertnstert.2023.07.025

[40] Clayton GL, Gonçalves A, Soares, Goulding N, Borges MC, Holmes MV, Davey Smith G, Tilling K, Lawlor DA, et al. A framework for assessing selection and misclassification bias in mendelian randomisation studies: An illustrative example between body mass index and COVID-19. BMJ. 2023;381: Article e072148. 37336561 10.1136/bmj-2022-072148 PMC10277657

[41] Day F, Karaderi T, Jones MR, Meun C, He C, Drong A, Kraft P, Lin N, Huang H, Broer L, et al. Large-scale genome-wide meta-analysis of polycystic ovary syndrome suggests shared genetic architecture for different diagnosis criteria. PLOS Genet. 2018;14(12): Article e1007813. 30566500 10.1371/journal.pgen.1007813 PMC6300389

[42] Lim SS, Hutchison SK, Van Ryswyk E, Norman RJ, Teede HJ, Moran LJ. Lifestyle changes in women with polycystic ovary syndrome. Cochrane Database Syst Rev. 2019;2019(3): Article CD007506. 10.1002/14651858.CD007506.pub4 PMC6438659 30921477

[43] Moran LJ, Brown WJ, McNaughton SA, Joham AE, Teede HJ. Weight management practices associated with PCOS and their relationships with diet and physical activity. Hum Reprod. 2017;32(3):669–678. 28069732 10.1093/humrep/dew348

[44] Dhar S, Mondal KK, Bhattacharjee P. Influence of lifestyle factors with the outcome of menstrual disorders among adolescents and young women in West Bengal, India. Sci Rep. 2023;13(1):12476. 37528155 10.1038/s41598-023-35858-2 PMC10393940

[45] Hemmert R, Schliep KC, Willis S, Peterson CM, Louis GB, Allen-Brady K, Simonsen SE, Stanford JB, Byun J, Smith KR. Modifiable life style factors and risk for incident endometriosis. Paediatric Perinat Epidemiol. 2019;33(1):19–25. 10.1111/ppe.12516 PMC6353682 30307628

[46] Southam L, Zeggini E. Twenty years of genome-wide association studies. Nature. 2025;641(8061):47–49. 40234607 10.1038/d41586-025-01128-6

[47] Tam V, Patel N, Turcotte M, Bossé Y, Paré G, Meyre D. Benefits and limitations of genome-wide association studies. Nat Rev Genet. 2019;20:467–484. 31068683 10.1038/s41576-019-0127-1

[48] Shrestha N, Kukkonen-Harjula KT, Verbeek JH, Ijaz S, Hermans V, Pedisic Z. Workplace interventions for reducing sitting at work. Cochrane Database Syst Rev. 2018;2018(12): Article CD010912. 10.1002/14651858.CD010912.pub5 PMC6517221 30556590

[49] Edwardson CL, Yates T, Biddle SJH, Davies MJ, Dunstan DW, Esliger DW, Gray LJ, Jackson B, O’Connell SE, Waheed G, et al. Effectiveness of the stand more AT (SMArT) work intervention: Cluster randomised controlled trial. BMJ. 2018;363: Article k3870. 30305278 10.1136/bmj.k3870 PMC6174726

[50] Leppe-Zamora J, Ramos-Fuster S, Muñoz-Monari B, Roa-Alcaino S, Sarmiento OL. The effect of computer prompt in breaks of sedentary behaviour among office workers: A systematic review and meta-analysis. Int J Behav Nutr Phys Act. 2025;22(1):75. 40514667 10.1186/s12966-025-01781-0 PMC12164069

[51] Lawlor DA. Commentary: Two-sample Mendelian randomization: Opportunities and challenges. Int J Epidemiol. 2016;45(3):908–915. 27427429 10.1093/ije/dyw127 PMC5005949

[52] Young DR, Sidell MA, Koebnick C, Saksvig BI, Mohan Y, Cohen DA, Wu TT. Longitudinal sedentary time among females aged 17 to 23 years. Am J Prev Med. 2019;56(4):540–547. 30773232 10.1016/j.amepre.2018.11.021 PMC6430668

[53] Liu Z-Y, Chen G-C, LaMonte MJ, Kamensky V, Evenson KR, Shadyab AH, Luo J, Allison M, Wild RA, Going SB, et al. Longitudinal transitions in sedentary behavior and physical activity in relation to all-cause and cause-specific mortality among postmenopausal women. GeroScience. 2025; 10.1007/s11357-025-01945-7 10.1007/s11357-025-01945-7 PMC13574740 41217670

[54] Hesketh KR, Baird J, Crozier SR, Godfrey KM, Harvey NC, Cooper C, van Sluijs E. Activity behaviors before and during pregnancy are associated with women’s device-measured physical activity and sedentary time in later parenthood: A longitudinal cohort analysis. J Phys Act Health. 2023;20(9):803–811. 37573030 10.1123/jpah.2022-0630 PMC7615174

[55] Li S, Du Y, Xu G, Bao W. Trends in sedentary behavior among US adults. JAMA. 2025;334(3):271–273. 40397460 10.1001/jama.2025.7220 PMC12096325

[56] Bull FC, Al-Ansari SS, Biddle S, Borodulin K, Buman MP, Cardon G, Carty C, Chaput J-P, Chastin S, Chou R, et al. World Health Organization 2020 guidelines on physical activity and sedentary behaviour. Br J Sports Med. 2020;54(24):1451–1462. 33239350 10.1136/bjsports-2020-102955 PMC7719906

[57] Mclaughlin M, Atkin AJ, Starr L, Hall A, Wolfenden L, Sutherland R, Wiggers J, Ramirez A, Hallal P, Pratt M, et al. Worldwide surveillance of self-reported sitting time: A scoping review. Int J Behav Nutr Phys Act. 2020;17(1):111. 32883294 10.1186/s12966-020-01008-4 PMC7469304

[58] Bycroft C, Freeman C, Petkova D, Band G, Elliott LT, Sharp K, Motyer A, Vukcevic D, Delaneau O, O’Connell J, et al. The UK Biobank resource with deep phenotyping and genomic data. Nature. 2018;562(7726):203–209. 30305743 10.1038/s41586-018-0579-z PMC6786975

[59] Constantinescu A-E, Mitchell RE, Zheng J, Bull CJ, Timpson NJ, Amulic B, Vincent EE, Hughes DA. A framework for research into continental ancestry groups of the UK Biobank. Hum Genomics. 2022;16(1):3. 35093177 10.1186/s40246-022-00380-5 PMC8800339

[60] Doherty A, Jackson D, Hammerla N, Plötz T, Olivier P, Granat MH, White T, van Hees V, Trenell MI, Owen CG, et al. Large scale population assessment of physical activity using wrist worn accelerometers: The UK Biobank study. PLOS ONE. 2017;12(2): Article e0169649. 28146576 10.1371/journal.pone.0169649 PMC5287488

[61] Venkatesh SS, Wittemans LBL, Palmer DS, Baya NA, Ferreira T, Hill B, Lassen FH, Parker MJ, Reibe S, Elhakeem A, et al. Genome-wide analyses identify 25 infertility loci and relationships with reproductive traits across the allele frequency spectrum. Nat Genet. 2025;57(5):1107–1118. 40229599 10.1038/s41588-025-02156-8 PMC12081293

[62] Kurki MI, Karjalainen J, Palta P, Sipilä TP, Kristiansson K, Donner KM, Reeve MP, Laivuori H, Aaviko M, Kaunisto MA, et al. FinnGen provides genetic insights from a well-phenotyped isolated population. Nature. 2023;613(7944):508–518. 36653562 10.1038/s41586-022-05473-8 PMC9849126

[63] The ENCODE Project Consortium. An integrated encyclopedia of DNA elements in the human genome. Nature. 2012;489(7414):57–74. 22955616 10.1038/nature11247 PMC3439153

[64] Hemani G, Zheng J, Elsworth B, Wade KH, Haberland V, Baird D, Laurin C, Burgess S, Bowden J, Langdon R, et al. The MR-base platform supports systematic causal inference across the human phenome. eLife. 2018;7: Article e34408. 29846171 10.7554/eLife.34408 PMC5976434

[65] Pulit T, Stoneman C, Morris AP, Wood AR, Glastonbury CA, Tyrell J, Yengo L, Ferreira T, Marouli E, Ji Y. Meta-analysis of genome-wide association studies for body fat distribution in 694,649 individuals of European ancestry. bioRxiv. 2018. 10.1101/304030 PMC6298238 30239722

[66] Sanderson E, Davey Smith G, Windmeijer F, Bowden J. An examination of multivariable Mendelian randomization in the single-sample and two-sample summary data settings. Int J Epidemiol. 2019;48(3):713–727. 30535378 10.1093/ije/dyy262 PMC6734942

[67] Hamilton FW, Hughes DA, Spiller W, Tilling K, Davey G. Non-linear Mendelian randomization: Detection of biases using negative controls with a focus on BMI, vitamin D and LDL cholesterol. Eur J Epidemiol. 2024;39(5):451–465. 38789826 10.1007/s10654-024-01113-9 PMC11219394

[68] Lawlor DA, Harbord RM, Sterne JAC, Timpson N, Davey Smith G. Mendelian randomization: Using genes as instruments for making causal inferences in epidemiology. Stat Med. 2008;27(8):1133–1163. 17886233 10.1002/sim.3034

[69] Bowden J, Davey Smith G, Burgess S. Mendelian randomization with invalid instruments: Effect estimation and bias detection through Egger regression. Int J Epidemiol. 2015;44(2):512–525. 26050253 10.1093/ije/dyv080 PMC4469799

[70] Yang Q, Sanderson E, Tilling K, Borges MC, Lawlor DA. Exploring and mitigating potential bias when genetic instrumental variables are associated with multiple non-exposure traits in Mendelian randomization. Eur J Epidemiol. 2022;37(7):683–700. 35622304 10.1007/s10654-022-00874-5 PMC9329407

[71] Yi W, Zhang M, Yuan X, Shi L, Yuan X, Sun M, Liu J, Cai H, Lv Z. A model combining testosterone, androstenedione and free testosterone index improved the diagnostic efficiency of polycystic ovary syndrome. Endocr Pract. 2023;29(8):629–636. 37225042 10.1016/j.eprac.2023.05.007

[72] Kowarik A, Templ M. Imputation with the *R* package VIM. J Stat Soft. 2016;74(7):1–16.

