# Supplementary material for: Sex-Stratified Genetic Analyses Mapping the Influences of Sedentary Behaviors and Physical Activity on Female Reproductive Health
Source: Research (Wash D C). 2026 Feb 16;9:1131. doi: 10.34133/research.1131 (PMC13183167; doi:10.34133/research.1131)
Supplement: Supplementary 1 — Fig. S1 Tables S1 to S18 [file research.1131.f1.zip › Supplemenatry Figure.pdf]

Sex-stratified genetic analyses mapping the influences of sedentary behaviours and physical activity on female reproductive health

Chongwen Shao, Qian Yang, Lanhui Huang et al

Supplementary Figure

Fig S1. Leave-one-out sensitivity analysis.

Sensitivity analysis by sequentially excluding each SNP to assess robustness of the causal estimate on (a) ectopic pregnancy, (b) endometriosis, (c) PCOS and (D)menorrhagia.

(a)

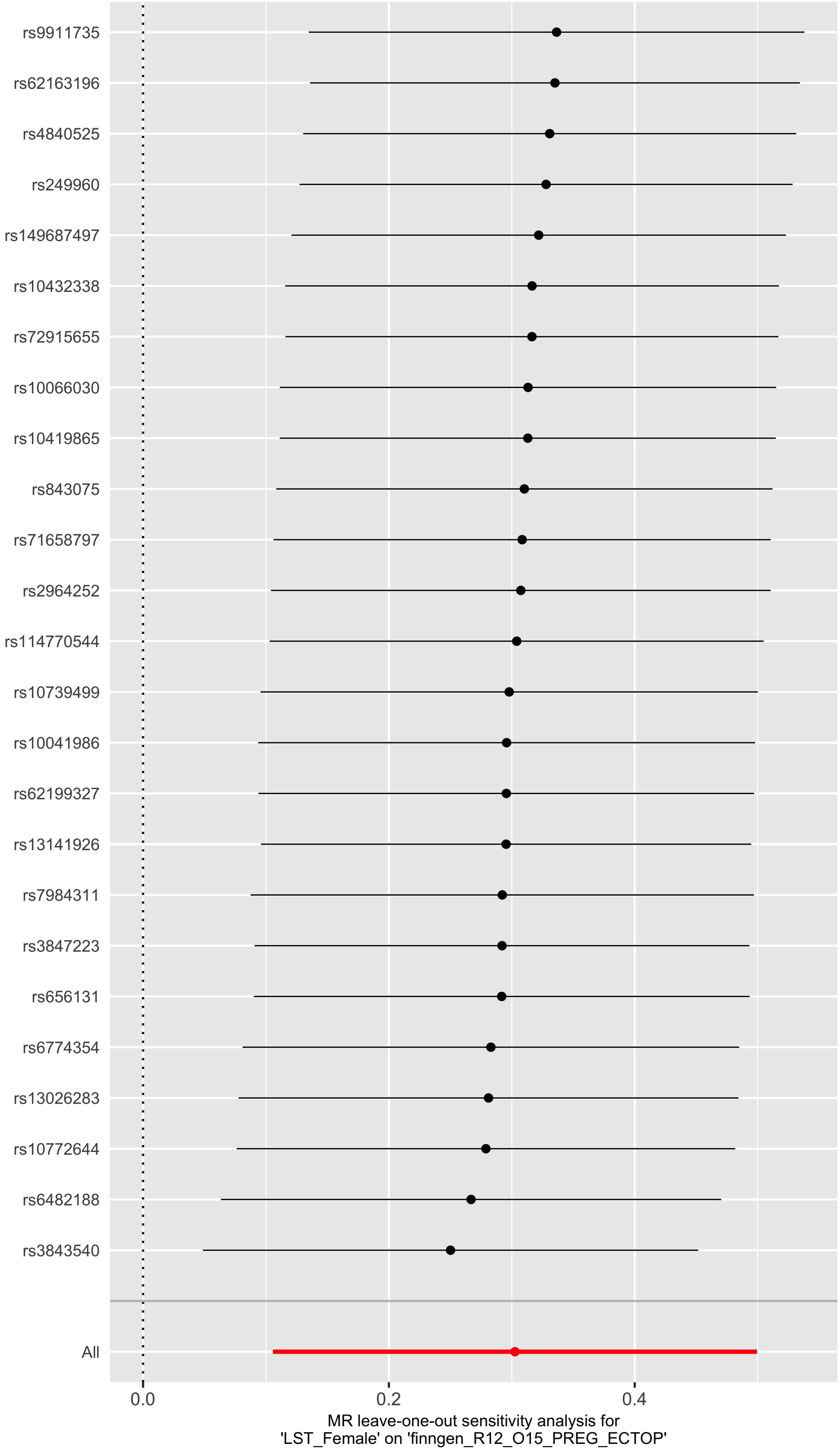

(b)

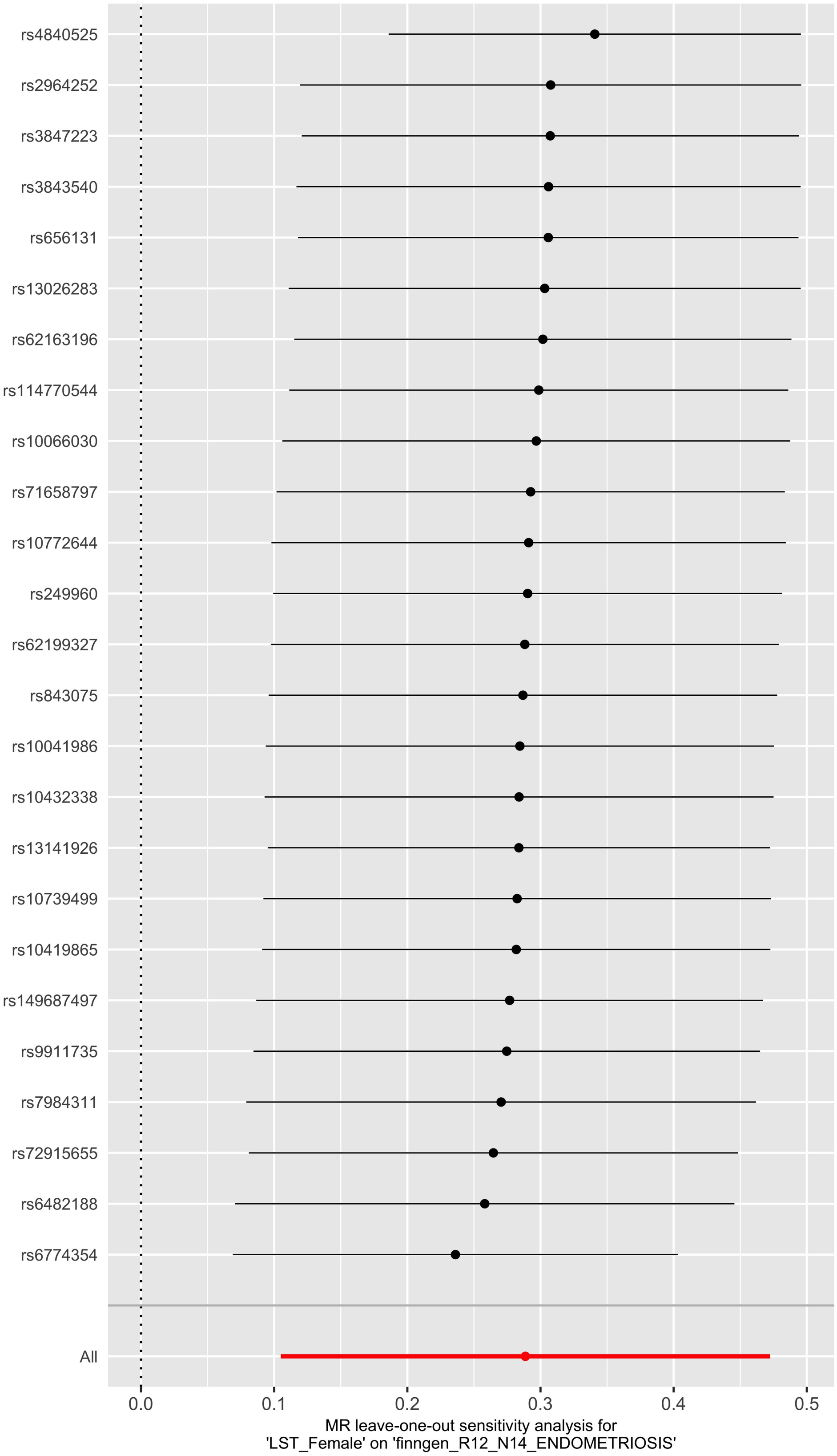

rs4840525
rs2964252
rs3847223
rs3843540
rs656131
rs13026283
rs62163196
rs114770544
rs10066030
rs71658797
rs10772644
rs249960
rs62199327
rs843075
rs10041986
rs10432338
rs13141926
rs10739499
rs10419865
rs149687497
rs9911735
rs7984311
rs72915655
rs6482188
rs6774354
All
0.0
0.1
0.2
0.3
0.4
0.5
MR leave-one-out sensitivity analysis for
'LST_Female' on 'finngen_R12_N14_ENDOMETRIOSIS'

(c)

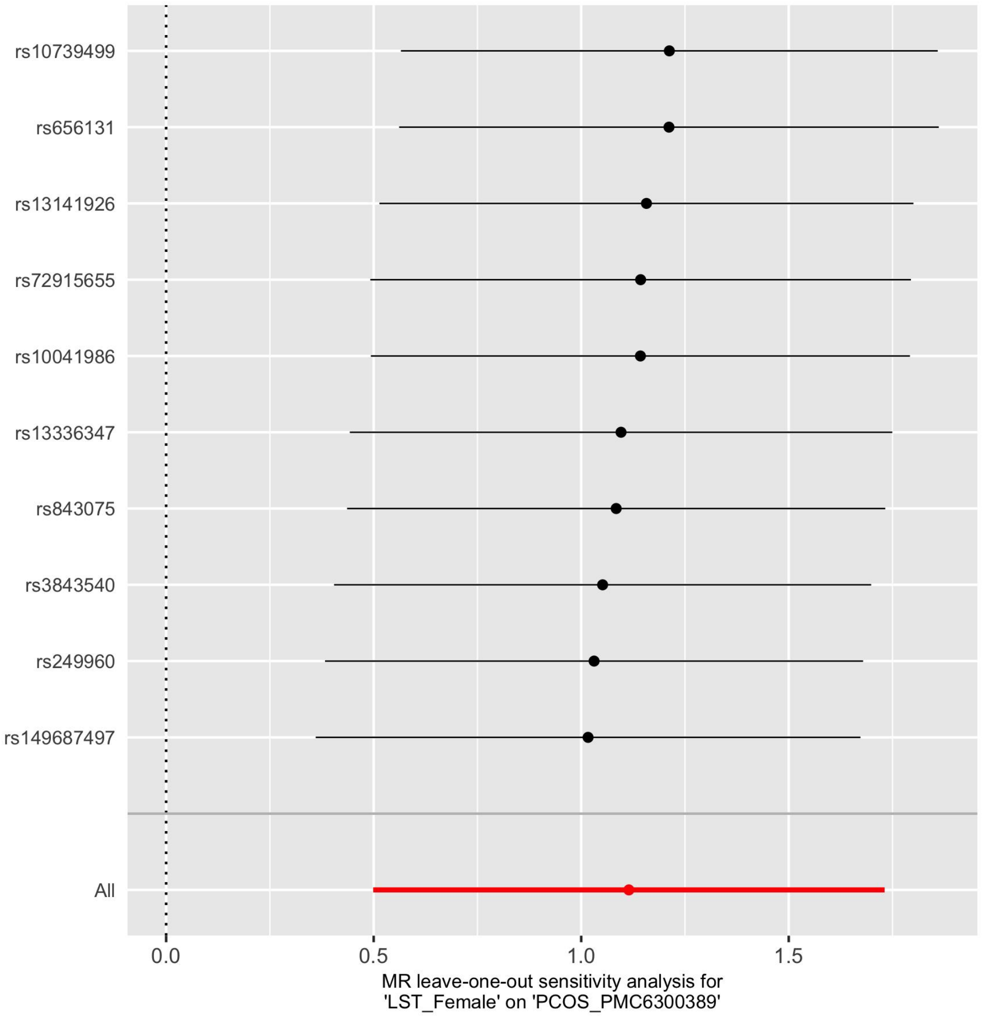

rs10739499
rs656131
rs13141926
rs72915655
rs10041986
rs13336347
rs843075
rs3843540
rs249960
rs149687497
All
0.0
0.5
1.0
1.5
MR leave-one-out sensitivity analysis for
'LST_Female' on 'PCOS_PMC6300389'

(d)

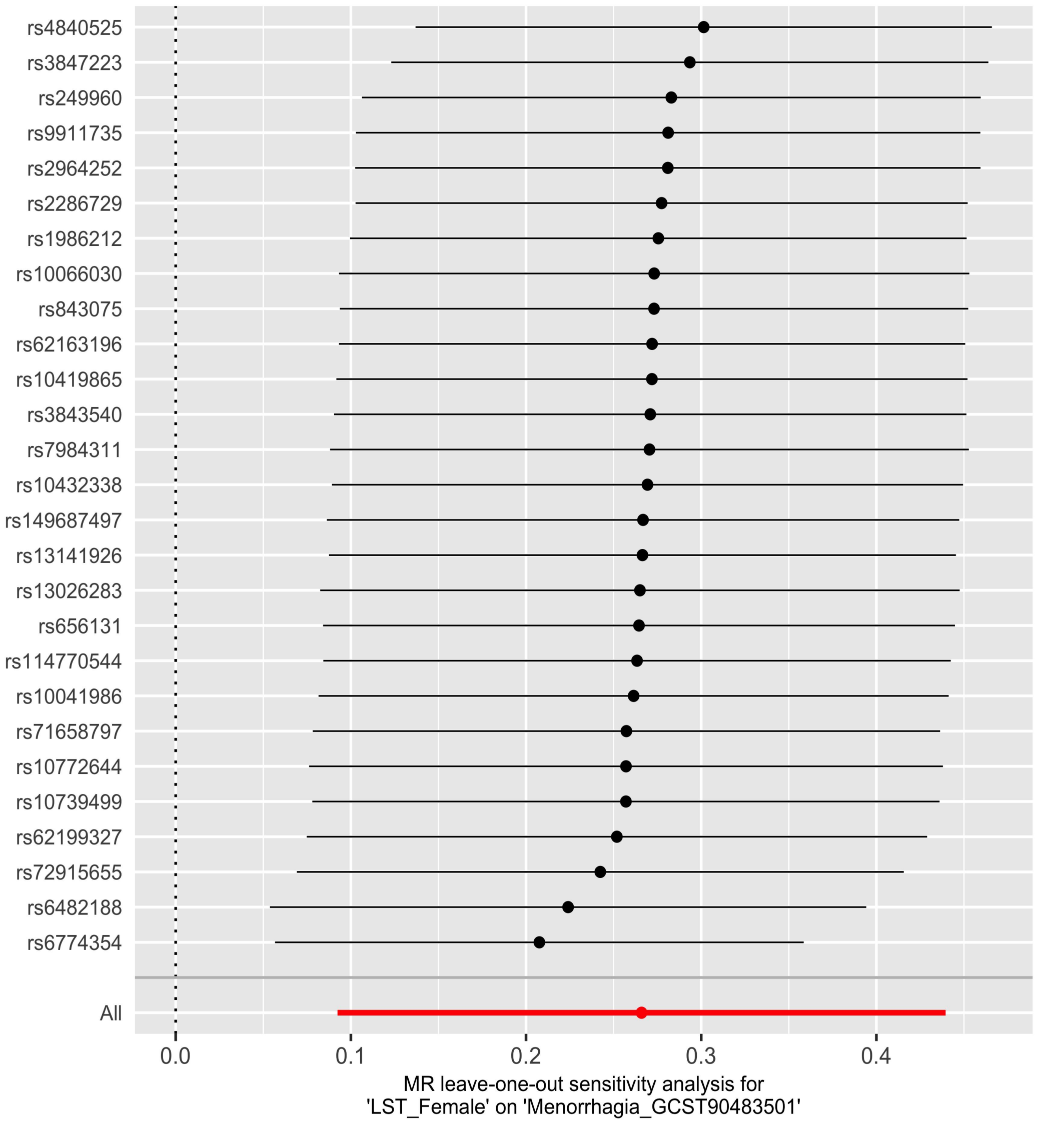
rs4840525
rs3847223
rs249960
rs9911735
rs2964252
rs2286729
rs1986212
rs10066030
rs843075
rs62163196
rs10419865
rs3843540
rs7984311
rs10432338
rs149687497
rs13141926
rs13026283
rs656131
rs114770544
rs10041986
rs71658797
rs10772644
rs10739499
rs62199327
rs72915655
rs6482188
rs6774354
All
0.0
0.1
0.2
0.3
0.4
MR leave-one-out sensitivity analysis for
'LST_Female' on 'Menorrhagia_GCST90483501'
